# Predicting Placenta Accreta Spectrum Disorders in a Cohort of Pregnant Patients in the North-East Region of Romania—Diagnostic Accuracy of Ultrasound and Magnetic Resonance Imaging

**DOI:** 10.3390/diagnostics12092130

**Published:** 2022-09-01

**Authors:** Raluca Maria Haba, Anda Ioana Pristavu, Maria-Luiza Cobzeanu, Alexandru Carauleanu, Ioana Sadiye Scripcariu, Ingrid Andrada Vasilache, Dorina Adelina Minciuna, Dragos Negru, Demetra Gabriela Socolov

**Affiliations:** 1 Department of Obstetrics and Gynecology, ‘Grigore T. Popa’ University of Medicine and Pharmacy, 700115 Iasi, Romania; 2 Obstetrics and Gynaecology Department, “Cuza Voda” Obstetrics and Gynecology University Hospital, 700115 Iasi, Romania; 3 Department of Radiology, Imaging University Hospital “Sf.Spiridon” Bd.Independentei 1, 700106 Iasi, Romania

**Keywords:** placenta accreta spectrum, ultrasonography, magnetic resonance imaging

## Abstract

**Background:** Placenta accreta spectrum (PAS) disorders are associated with high mortality and morbidity due to postpartum hemorrhage, hysterectomy, and organ injury, and a multidisciplinary team is required for an individualized case management. In this study, we assessed the diagnostic and prognostic accuracy of the most important ultrasonographic (US) and magnetic resonance imagining (MRI) markers for PAS disorders. **Material and Methods:** The study included 39 adult pregnant patients with at least one previous cesarean delivery and both US and MRI investigations for placenta previa evaluated at the tertiary maternity hospital ‘Cuza Voda’, Iasi, between 2019 and 2021. The following US signs were evaluated: intra-placental lacunae, loss of the retroplacental hypoechoic zone, myometrial thinning < 1 mm, bladder wall interruption, placental bulging, bridging vessels, and the hypervascularity of the uterovesical or retroplacental space. The MRI signs that were evaluated were intra-placental dark T2 bands, placental bulging, loss of the retroplacental hypointense line on T2 images, myometrial thinning, bladder wall interruption, focal exophytic placental mass, and abnormal vascularization of the placental bed. **Results:** The US and MRI signs analyzed in our study presented adequate sensitivities and specificities for PAS, but no sign proved to be a useful predictor by itself. The presence of three or more US markers for accretion was associated with a sensitivity of 84.6.6% and a specificity of 92.3% (*p* < 0.001). The presence of three or more MRI signs supplemented these results and were associated with a sensitivity of 92.3% and a specificity of 61.5% for predicting PAS (*p* < 0.001). Moreover, US and MRI findings were correlated with FIGO grading and severity of PAS. **Conclusions:** Even though no US or MRI finding alone can predict PAS with high sensitivity and specificity, our study proves that the presence of three or more imagistic signs could significantly increase the diagnostic accuracy of this condition. Furthermore, US and MRI could be useful tools for evaluating prognostic and perinatal planning.

## 1. Introduction

Placenta accreta spectrum (PAS) disorders reunite three pathological entities characterized by the abnormal adhesion and invasion of trophoblastic tissue into the myometrium and uterine serosa [1,2]. The particularity of these disorders is determined by the progression from less severe stages such as placenta accreta, where placental villi adhere to the myometrium, to placenta increta, characterized by the invasion of placental villi into the myometrium, and placenta percreta, where placental invasion reaches the uterine serosa or the surrounding anatomical structures [3]. 

Damage to the endometrium-myometrial interface appears to be the main determinant of PAS disorders [1]. Due to increased cesarean delivery rates worldwide [4,5,6,7], the PAS disorders incidence is expected to follow an ascending trend. A 20-year analysis of cases with abnormal placentation by Wu et al. described an increase of about four-fold, from 0.08% to 0.3%, in the incidence of placenta accreta [8]. Besides cesarean delivery, several surgical procedures that alter the integrity of the uterus, such as suction curettage, endometrial ablation and resection, hysteroscopy, and adhesiolysis were identified [9,10]. 

PAS disorders are associated with high mortality and morbidity, and a multidisciplinary team is required for individualized case management. Postpartum hemorrhage, hysterectomy, and organ injury are cited as serious complications associated with PAS disorders [11,12]. A thorough prenatal assessment of cases with at least one cesarean section or personal history of uterine surgery and a low-lying placenta or placenta previa demonstrated with ultrasound examination could identify women at high risk of developing PAS disorders [13,14,15,16]. 

Ultrasound assessment of PAS markers can be performed as early as in the first trimester, and special attention should be offered to the relationship between the gestational sac and previous cesarean scar [17]. It appears that a low implantation of the gestational sac within or in close proximity to a cesarean scar is strongly associated with the presence of placenta accreta in the last trimester of pregnancy [18]. Moreover, a meta-analysis conducted by D’Antonio et al., reported the detection of at least one ultrasound sign suggestive of PAS in 91.4% of women with confirmed PAS in the first trimester. Among the ultrasound signs, the authors included low implantation of the gestational sac, reduced myometrial thickness, and lacunae [19]. 

As pregnancy progresses, serial ultrasound examinations performed by skilled obstetricians should be offered to pregnant patients at a high risk for PAS disorders. The American College of Obstetricians and Gynecologists (ACOG)/Society for Maternal-Fetal Medicine (SMFM) suggest the timing for ultrasound examinations at approximately 18–20, 28–30, and 32–34 weeks of gestation in asymptomatic patients [14].

Several ultrasound signs and scoring systems have been proposed for the screening of PAS disorders in the second and third trimester of pregnancy. The most important set of signs for abnormal placentation was described by the European Working Group on Abnormally Invasive Placenta [20] and included loss of the clear zone, myometrial thinning < 1 mm, placental lacunae, bladder wall interruption, placental bulge, exophytic mass, subplacental and/or uterovesical hypervascularity, placental lacunae feeder vessels, and bridging vessels. The prenatal ultrasound appears to have a high accuracy for the diagnosis of PAS in high-risk pregnant women, with a sensitivity of 91% and a specificity of 97% [21]. 

The placenta accreta index, developed by Rac et al., combines the number of cesarean deliveries, placental location, and ultrasound markers such as reduced myometrial thickness, intraplacental lacunae, and bridging vessels in order to predict PAS disorders with an area under the receiver operating characteristic (ROC) curve of 0.87 [22,23]. Another scoring system, proposed by Cali et al., has the advantage of correspondence with the International Federation of Gynecology and Obstetrics (FIGO) clinical grading system and uses various ultrasound signs that could predict postpartum complications [24]. Further studies will be needed to evaluate the performance of these scoring systems regarding the risk stratification for pregnant women at a high risk for PAS disorders. 

Magnetic resonance imaging can be used as a diagnostic tool for PAS disorders in specific situations: significant maternal obesity, difficult or uncertain ultrasound diagnosis, and the evaluation of placental invasion [25,26]. Similar to the ultrasound signs, some imaging markers have been cited for MRI evaluation and are represented by uterine bulging and loss of normal uterine contour, heterogeneous signal intensity within the placenta, dark intraplacental bands on T2-weighted images, abnormal placental vascularity, focal thinning of the myometrial and interruption of the junctional zone (focal interruptions in the myometrial wall), tenting of the bladder, and direct visualization of the invasion of near-by organs. It has been demonstrated that these signs are able to detect placenta accreta, increta, and percreta with a sensitivity of 94.4%, 100%, and 86.5% and a specificity of 98.8%, 97.3%, and 96.8% [25].

A significant number of patients with PAS disorders remain undiagnosed until surgery [27]. Consequently, standardized protocols for the prenatal assessment of high-risk patients are needed to improve the detection rate [28]. Moreover, a correct prenatal diagnosis of PAS disorders could allow for the counselling of future mothers regarding the risks associated with their pregnancy. 

In this study, we aimed to retrospectively assess the diagnostic accuracy of the most important sonographic and magnetic resonance markers for PAS disorders in a cohort of patients with previous cesarean delivery and placenta previa. Furthermore, we wanted to highlight the possible association between imaging parameters and clinical grades of PAS disorders. 

## 2. Materials and Methods

Adult pregnant patients (>18 years old) with placenta previa and at least one previous cesarean delivery, who underwent both ultrasonographic and MRI investigations for suspicion of PAS, were included in this retrospective single-center study at a tertiary maternity hospital ‘Cuza Voda’, Iasi, between 1 January 2019, and 1 November 2021. Exclusion criteria consisted of patients who had ectopic pregnancies, first- and second-trimester pregnancy loss, patients who failed to participate in all the study visits, patients who were unable to offer their informed consent, and patients who could not be assessed by ultrasound and MRI. 

Ethical approval for this study was obtained from the Institutional Ethics Committees of University of Medicine and Pharmacy ‘Grigore T. Popa’ (No. 189/25 May 2022), and ‘Cuza Voda’ Maternity Hospital, Iasi (No. 2461/25 February 2022). Informed consent was obtained from all participants included in the study. All methods were carried out in accordance with relevant guidelines and regulations. 

Medical records of 39 patients were systematically reviewed, and data were obtained. The following variables were recorded: maternal age, gestation, parity, BMI (body mass index), smoking status, patient medical history, gestational age at birth, child APGAR score, and birth weight. Furthermore, for the PAS positive group we evaluated parameters related to surgical outcome such as: total or subtotal hysterectomy, urinary bladder injury, ureteral injury, hypogastric artery ligation, necessity of blood transfusions and number of days of hospitalization. All patients underwent ultrasound scans performed by a maternal-fetal specialist using Voluson E8 and E10 Expert machines (General Electric Healthcare, Zipf, Austria) equipped with a 2–5 MHz transabdominal convex transducer and a 4–9 MHz transvaginal transducer. The following ultrasound signs were evaluated: intra-placental lacunae, loss of the retroplacental hypoechoic zone, myometrial thinning < 1 mm, bladder wall interruption, placental bulging, bridging vessels, and the hypervascularity of the uterovesical or retroplacental space. 

MRI examinations were performed between 28 and 35 weeks of gestation using a 1.5 Tesla scan system (Achieva, Philips, Amsterdam, The Netherlands) with a surface phased-array coil. All the examinations included the following sequences T2 weighted: ultrafast single-shot sequences and turbo spin echo (SShTE) and SShTE + Spectral Presaturation with Inversion Recovery (SPIR) in all 3 planes (axial, coronal and sagittal plane) and diffusion-weighted imaging (DWI) sequence in the sagittal plane. The SShTE sequences were obtained using the ratio between repetition time and the echo time (TR/TE) 516/80, field of view (FOV) 450 × 362, acquisition matrix 376 × 258, flip angle 90°, slice thickness 5 mm, and interslice gap 0.5 mm. The DWI sequences were obtained using FOV 280 × 200, acquisition matrix 188 × 133, slice thickness 5 mm, and interslice gap 1 mm. SShTE sequences are relatively resistant to maternal and fetal motion artifacts and provide reasonable differentiation between the placental tissue and underlying myometrium. All the sequences have been acquired during maternal breath holding. An intravenous contrast medium was not used. 

All the MRI examinations of the patients in our study underwent the evaluation of seven signs suggestive for PAS disorders: intra-placental dark T2 bands, placental bulging, loss of retroplacental hypointense line on T2 images, myometrial thinning, bladder wall interruption, focal exophytic placental mass, and abnormal vascularization of the placental bed. All US and MRI signs are exemplified in Figures S1 and S2.

All patients were scheduled for caesarean delivery between 34 and 36 weeks +6 days, based on their clinical status. After the fetus was delivered, uterine massage and controlled cord traction under administration of Carbetocin were performed in order to expel the placenta. If the placental removal was successful, the patients were defined as PAS-negative. If there were clinical signs that placenta had reached the serosa, there were no attempts to detach it manually. A total hysterectomy with the placenta in situ was conducted in the event of serosal invasion or failure to remove the placenta, and the specimen was sent for histopathologic analysis. These patients formed the PAS-positive group. None of our patients with clinical signs of abnormally invasive placenta during surgery received a conservative management or uterine segmental resection. All surgical specimens underwent histological examination to establish the diagnosis of placenta accreta, increta or percreta.

The patients were also categorized into four groups based on histological evaluation and/or surgical grading according to the FIGO guidelines [15]: group 1 (grade I/placenta accreta), group 2 (grade II/placenta increta), group 3 (grade IIIa/placenta percreta limited to the serosa), and group 4 (grade IIIb/placenta percreta with bladder invasion). In our cohort of patients, none had been diagnosed with grade IIIc placenta percreta with the invasion of organs other than the bladder. 

**Figure 1:**
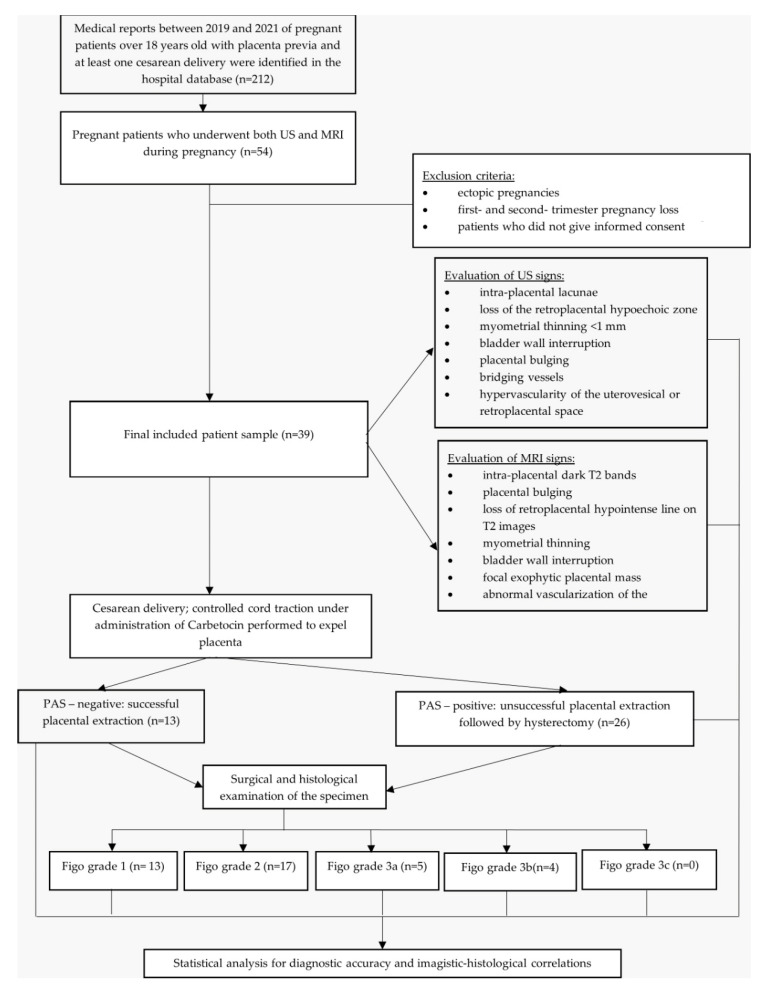
Flow chart of the study design.

Student *t*-test was used to compare the continuous variables between the PAS-positive group and the PAS-negative group. The correlation between variables in our study was measured using the Pearson correlation coefficient for continuous variables or Spearman correlation coefficient for nonparametric variables. A diagnostic accuracy analysis and a multivariate logistic regression of the available data were performed using the SPSS software (version 28.0.1, IBM Corporation, Armonk, NY, USA). A *p* value less than 0.05 was considered statistically significant. The study design algorithm is presented in Figure 1.

## 3. Results

During the 3 years of investigation, 39 pregnant patients were included in our study. The patient’s characteristics and risk factors for PAS disorders were compared between the PAS-positive group (n = 26) and the PAS-negative group (n = 13), and the results are presented in Table 1. Only the number of previous cesarean deliveries (*p* = 0.032), the body mass index (BMI) (*p* = 0.044) and number of days of hospitalization (*p* < 0.01) were significantly different between the two groups. There were no significant differences when analyzing neonatal outcomes (APGAR score and birth weight) between the two groups. 

In the PAS-positive group, 20 patients (76.9%) underwent total hysterectomy, while 6 (23.1%) patients underwent subtotal hysterectomy. The most common adverse outcome during hysterectomy was urinary bladder injury (14 cases), followed by hypogastric artery ligation (10 patients), ureteral injury (7 cases) and necessity of blood transfusion (7 cases), data shown in Table 2.

We evaluated the diagnostic accuracy of seven ultrasound signs related to PAS disorders, and the results are presented in Table 3. The PAS-positive patients presented significantly a higher number of sonographic signs compared to PAS-negative women (mean ± SD: 4.65 ± 1.41 versus 2.08 ± 1.11, *p*< 0.001). The most frequently identified ultrasound signs in PAS-positive patients were loss of retroplacental clear zone (n = 24), which had a sensibility (Se) of 92.3%, and a specificity (Sp) of 46.1%, placental lacunar spaces (n = 24; Se-92.3%, Sp-76.9%), and myometrial thinning < 1 mm (n = 24; Se-92.3%, Sp-76.9%). The highest accuracy for ultrasound markers was 0.87 for placental lacunae and myometrial thinning, followed by 0.84 for bridging vessels and 0.76 for the loss of the retroplacental clear zone. The presence of three or more US signs was associated with a sensitivity of 84.6%, a specificity of 92.3%, and a diagnostic accuracy of 0.87 for PAS prediction.

The results from the multivariate analysis of ultrasound signs indicated a significant association between placental lacunar spaces, myometrial thinning, and grade I and II PAS (*p* = <0.001 and *p* = 0.024); between the loss of retroplacental clear zone, grade I PAS (*p* = 0.005), and grade II PAS (*p* = 0.047); between bladder wall interruption, grade IIIa PAS (*p* = 0.028), and grade IIIb PAS (*p* = 0.005); between hypervascularity of the uterovesical or retroplacental space, grade IIIa PAS (*p* = 0.011) and grade IIIb PAS (*p* = 0.002); and between placental bulging and grade IIIb PAS (*p* < 0.001). The complete results are presented in Table 4. 

We further evaluated the diagnostic accuracy of seven magnetic resonance imaging signs related to PAS disorders, and the results are presented in Table 5. The PAS-positive patients presented a significantly higher number of MRI signs compared to PAS-negative women (mean ± SD: 4.92 ± 1.6 versus 1.15 ± 1.28, *p* < 0.001). The most frequently identified MRI sign in PAS-positive patients was myometrial thinning < 1 mm (n = 26), which had a Se of 100% but a poor specificity. This sign was followed by loss of retroplacental hypointense line in T2 images (n = 25; Se-96.1%, Sp-61.5%) and intraplacental dark T2 bands (n = 24; Se-92.3%, Sp-38.4%). All MRI signs, except for miometrial thinning and focal exophytic placental mass, were significantly different between the two groups. The highest accuracy of MRI signs was 0.84 for the loss of retroplacental hypointense line on T2 images, followed by 0.74 for intraplacental dark T2 bands and 0.71 for abnormal vascularization of the placental bed. The presence of three or more MRI signs was associated with a sensitivity of 92.3%, a specificity of 61.5%, and a diagnostic accuracy of 0.82 for PAS prediction.

The results from the multivariate analysis of MRI signs indicated a significant association between intraplacental dark T2 bands, grade I PAS (*p* < 0.001), and grade II PAS (*p* = 0.010); between loss of retroplacental hypointense line on T2 images, grade I PAS (*p* < 0.001), and grade II PAS (*p* = 0.025); between placental bulging, grade IIIa (*p* = 0.019), and grade IIIb (*p* < 0.001); and between bladder wall interruption, grade IIIa (*p* = 0.006), and grade IIIb (*p* = 0.028). The complete results are presented in Table 6.

In Table 7, we summarize the relative incidence and the true predictive value of all US and MRI signs presented above.

Analyzing the correlation between the number of ultrasonography and MRI signs and an adverse outcome we found that the presence of more than 3 US or 3 MRI signs are associated with a necessity of total hysterectomy (*p* < 0.001) and an increased number of hospitalization days (*p* < 0.05). Moreover, the presence of 3 or more MRI signs in patients with PAS was correlated with intraoperative bladder injury (*p* = 0.04) and hypogastric artery ligation (*p* = 0.03) (data shown in Table 8).

## 4. Discussion

The prenatal recognition of PAS disorders is vital for individualizing patient management and counselling. Currently, ultrasound remains the first-line modality for the screening and diagnosis of PAS disorders in pregnant women due to the reduced cost, its accessibility, and its reproducibility. Even though MRI is not routinely recommended for all cases of PAS, it can help the diagnosis due to increased specificity (98% vs. 95% for US) and sensitivity (90% vs. 77% for US) [29,30]. Moreover, MRI can complement US examination to guide the prenatal and intraoperative management in specific cases. 

Our study evaluated the main ultrasound and MRI signs cited in literature to be associated with PAS disorders in terms of diagnostic accuracy. Furthermore, we tried to evaluate if the association of multiple signs can improve the diagnostic power and refine the prognostics of the disease. 

In our cohort of patients, all the evaluated ultrasound signs were significantly associated with PAS-positive women, but the highest diagnostic accuracy was presented by myometrial thinning (87%) and placental lacunar spaces (87%), followed by the presence of bridging vessels (84%). The maximum sensitivity (92.3%) was held by the loss of the retroplacental clear zone, placental lacunar spaces, and myometrial thinning, while the maximum specificity was held by bladder wall interruption (100%) followed by bridging vessels and hypervascularity of the uterovesical or retroplacental space (92.3%).

In a 3-year study which enrolled 314 patients, Pilloni et al. found a similar sensitivity (83%) for the loss of the retroplacentar clear zone [31]. Similarly, Jauniaux et al., after analyzing over 83 studies that evaluated over 1078 cases of PAS, identified the loss of the retroplacental clear zone and the presence of placental lacunae as the most prevalent US signs (98% and 96.1%) in patients with accretion [32]. In a different study, Gulati et al. found that Doppler US could be used as an important tool for the detection of bridging vessels, which had a high specificity (93.5%) for PAS [33], which was comparable to our study (Sp-92.3%). On the other hand, in our study, placental bulging presented a very low sensitivity (30.7%) and a maximum specificity, while other authors reported good sensitivity (91.7%) and poorer specificity (76.9%) [34].

The highest accuracy of MRI signs was held by the loss of retroplacental hypointense line on T2 images (84%), followed by intraplacental dark T2 bands (74%) and abnormal vascularization of the placental bed (71%). The maximum sensitivity was held by myometrial thinning, which was followed by a loss of retroplacental hypointense line on T2 images (96.1%) and intraplacental dark T2 bands (92.3%). In a similar study, Ishibashi et al. reported that myometrial thinning had a sensitivity of 87.5% for predicting PAS, while intraplacental T2 dark bands had a sensitivity of 75% [35]. 

In our study, the maximum specificity was held by placental bulging, together with bladder wall interruption and focal exophytic placental mass. The importance of the placental bulge as a diagnostic tool for both US and MRI was highlighted, especially as a predictor for myometrial invasion [36]. The diagnostic value of placental bulging was higher in MRI investigations (Se-83.33%, Sp-77.77%) than in US (Se-55.55%, Sp-100%), however, with no significant statistical difference between the two. This may be since MRI provides a higher soft-tissue contrast, which results in better visualization of anatomical details. This is extremely valuable, especially in obese patients, patients with restricted FOV, lack of a coronal view and lack of visualization of parametrial invasion. Moreover, MRI is especially useful for the evaluation of posteriorly located placentas where US imaging alone has a very low diagnostic accuracy [37].

The placenta accreta spectrum is a polymorphic condition with multiple phenotypes, and as a result, a singular imagistic finding cannot function as a diagnostic tool. The US and MRI signs analyzed in our study showed adequate sensitivities and specificities for PAS. No sign proved to be a useful predictor by itself. In the case of US, placental lacunar spaces and myometrial thinning both had good sensitivity but a lower specificity (Se-92.3% and Sp-76.9%). Regarding MRI signs, the loss of retroplacental hypointense line on T2 images also showed a good sensitivity (96.1%) but a poor specificity (61.5%). However, according to the analysis of the presence of more than one US or MRI predictor for PAS, the diagnostic capacities increased significantly. In our study, the presence of three or more US markers for accretion was associated with a sensitivity of 84.6% and a specificity of 92.3% (*p* < 0.001). The presence of three or more MRI signs supplemented these results and were associated with a sensitivity of 92.3% and a specificity of 61.5% for predicting PAS (*p* < 0.001). The usage of more than one imagistic sign or clinical and imagistic scores for predicting accretion was also proposed by multiple authors. Correspondingly, Gao et al. imagined an imagistic score based on US examination, which presented a sensitivity of 83.3% and a specificity of 85.7% [38]. Likewise, Romeo et al., evaluating 70 patients diagnosed with PAS, proposed a diagnostic algorithm incorporating clinical risk factors, US and MRI findings [39].

In addition to diagnostic capabilities, the imaging of PAS plays an important role in the prognosis of a more severe condition or adverse outcomes. The FIGO classification can be used to assess the pathological severity, with FIGO III patients being associated with the highest incidence of adverse events in the PAS population. In our study, US findings such as bladder interruption, the presence of bridging vessels, and hypervascularity of the uterovesical or retroplacental space were significantly correlated with the presence of either FIGO Grade IIIa and Grade IIIb of PAS (*p* < 0.05). Similarly, MRI signs of percretization such as placental bulging, bladder wall interruption, and focal exophytic placental mass were also associated with FIGO Grade IIIa and Grade IIIb of PAS (*p* < 0.05). 

Our results complement several other studies that reported bladder wall interruption and uterovesical interruption as predictors of a worse outcome in PAS patients [24,40]. In the case of MR imaging, similar percretization descriptors to those used in our study were associated with adverse events in several studies investigating PAS. However, other markers such abnormal vascularity and low-intensity bands on T2-weighted images were significantly correlated with FIGO III patients, which was not validated in our investigation [41,42]. Moreover, our study also found correlations between increased number of US and MRI signs and an adverse outcome of surgery such as urinary bladder injury, hypogastric artery ligation and increased number of hospitalization days. These results reinforce the hypothesis that prenatal imaging evaluation could be used for assessing the risk of PAS patients and adequate preoperative planning. 

Our study has several limitations: a small sample size, which may have contributed to the lack of statistical significance for many of the observed differences, and the exclusion of patients without prior CS even if they had a history of other uterine surgeries. The strengths of this study are determined by the use of standardized imaging parameters for the diagnosis of PAS disorders and standardized classification for PAS severity, making it the first study of this design in our country. 

## 5. Conclusions

The present study confirms the utility of prenatal ultrasonographic and magnetic resonance imaging for the diagnosis of placenta accreta spectrum disorders. Even though no US or MRI finding alone can predict PAS with high sensitivity and specificity, our study proves that the presence of three or more imagistic signs could significantly increase the diagnostic accuracy of this condition. Furthermore, US signs, such as bladder interruption, the presence of bridging vessels, and hypervascularity of the uterovesical or retroplacental space, and MRI signs, such as placental bulging, bladder wall interruption, and focal exophytic placental mass, were significantly associated with more severe forms of PAS. Therefore, comprehensive imaging evaluation during gestation could be helpful for predicting the severity of PAS and could be an essential tool for preoperative planning and reducing adverse events.

## Figures and Tables

**Table 1:** Patients’ characteristics and risk factors for PAS segregated between PAS-positive and PAS-negative groups.

Patient’s Characteristics	Pas Positive (n = 26)	Pas Negative (n = 13)	*p* Value
Age (years, mean ± SD)	32 ± 3.7	31.08 ± 4.17	0.49
BMI (kg/m2, mean ± SD)	30.26 ± 3.49	28.13 ± 1.59	0.044
Current smoker (%)	13 (50%)	4 (30.76%)	0.25
Number of gestations (mean ± SD)	3.38 ± 2.17	2.54 ± 1.26	0.20
Parity (mean ± SD)	2.96 ± 2.14	2.23 ± 0.72	0.24
Previous CS (mean ± SD)	2.3 ± 1.54	1.31 ± 0.63	0.032
Gestational age at delivery (weeks, mean ± SD)	35.77 ± 2.1	36.15 ± 1.21	0.55
Birth weight (grams, mean ± SD)	2804.6 ± 482.4	2963 ± 307.3	0.219
APGAR score (mean ± SD)	6.9 ± 1.7	6.7 ± 1.1	0.868
Hospitalization (days, mean ± SD)	4.38 ± 1.75	7.77 ± 2.55	<0.01

**Table 2:** Intraoperative outcome in the PAS-positive group.

Intraoperative Outcome	Characteristics	
	Yes (No, %)	No (No, %)
Total hysterectomy	20 (76.9%)	6 (23.1%)
Subtotal hysterectomy	6 (23.1%)	20 (76.9%)
Intraoperative urinary bladder injury	14 (53.8%)	12 (46.2%)
Intraoperative ureteral injury	7 (26.9%)	19 (73.1%)
Hypogastric artery ligation	10 (38.5%)	16 (61.5%)
Necessity of blood transfusion	7 (26.9%)	19 (73.1%)

**Table 3:** Diagnostic accuracy of ultrasound signs related to PAS disorders.

Ultrasound Signs	Pas Positive (n = 26)	Pas Negative (n = 13)	Se	Sp	Accuracy	PPV	NPV	*p* Value
Placental lacunar spaces	24	3	92.3% (24/26)	76.9% (7/13)	0.87	88.8% (24/27)	83.3% (10/12)	<0.001
Loss of retroplacental clear zone	24	7	92.3% (24/26)	46.1% (7/13)	0.76	77.4% (24/31)	75% (6/8)	0.005
Bladder wall interruption	14	0	53.8% (14/26)	100% (13/13)	0.69	100% (14/14)	52% (13/25)	0.001
Myometrial thinning < 1 mm	24	3	92.3% (24/26)	76.9% (10/13)	0.87	88.8% (24/27)	83.3% (10/12)	<0.001
Placental bulging	8	0	30.7% (8/26)	100% (13/13)	0.53	100% (8/8)	41.9% (13/31)	0.025
Bridging vessels	21	1	80.7% (21/26)	92.3% (12/13)	0.84	95.4% (21/22)	70.5% (12/17)	<0.001
Hypervascularity of the uterovesical or retroplacental space	11	1	42.3% (11/26)	92.3% (12/13)	0.58	91.6% (11/12)	44.4% (12/27)	0.027
≥3 US signs present	22	1	84.6% (22/26)	92.3% (12/13)	0.87	95.6% (22/23)	75% (12/16)	<0.001

Table legend: Se—sensibility; Sp—specificity; PPV—positive predictive value; NPV—negative predictive value.

**Table 4:** Multivariate logistic regression for the association between ultrasound signs and FIGO grading of PAS disorders.

Ultrasound Signs	Grade I	*p* Value	Grade II	*p* Value	Grade IIIa	*p* Value	Grade IIIb	*p* Value
Placental lacunar spaces (OR+ CI 95%)	0.025 (0.004–0.173)	<0.001	6.250 (1.145–34.123)	0.024	0.647 (0.505–0.829)	0.110	0.657 (0.517–0.835)	0.159
Loss of retroplacental clear zone (OR+ CI 95%)	0.097 (0.16–0.593)	0.005	7.467 (0.819–68.1)	0.047	1.037 (0.1–10.806)	0.976	0.771 (0.644–0.924)	0.284
Myometrial thinning < 1 mm (OR+ CI 95%)	0.58 (0.11–0.312)	<0.001	6.250 (1.145–34.123)	0.024	1.913 (0.191–19.198)	0.576	0.657 (0.517–0.835)	0.159
Bladder wall interruption (OR+ CI 95%)	0.083 (0.009–0.737)	0.009	0.602 (0.157–2.31)	0.458	9.6 (0.951–96.922)	0.028	0.286 (0.169–0.482)	0.005
Bridging vessels (OR+ CI 95%)	0.20 (0.002–0.190)	<0.001	2.88 (0.755–10.987)	0.117	0.5 (0.357–0.7)	0.035	0.514 (0.373–0.71)	0.063
Placental bulging (OR+ CI 95%)	0.692 (0.536–0.895)	0.025	0.356 (0.062–2.043)	0.234	3.111 (0.423–22.866)	0.248	0.114 (0.45–0.287)	< 0.001
Hypervascularity of the uterovesical or retroplacental space (OR+ CI 95%)	0.114 (0.013–1.009)	0.027	0.310 (0.068–1.4)	0.119	13 (1.265–133.635)	0.011	0.229 (0.124–0.42)	0.002

**Table 5:** Diagnostic accuracy of MRI signs related to PAS disorders.

MRI Signs	Pas Positive (n = 26)	Pas Negative (n = 13)	Sp	Sp	Accuracy	PPV	NPV	*p* Value
Intraplacental dark T2 bands	24	8	92.3% (24/26)	38.4% (5/13)	0.74	75.% (24/32)	71.4% (5/7)	0.018
Placental bulging	8	0	30.7% (8/26)	100% (13/13)	0.53	100% (8/8)	41.9% (13/31)	0.025
Loss of retroplacental hypointense line on T2 images	25	5	96.1% (25/26)	61.5% (8/13)	0.84	83.3% (25/30)	88.8% (8/9)	<0.001
Myometrial thinning < 1 mm	26	12	100% (26/26)	7.69% (1/13)	0.69	68.4% (26/38)	100% (1/1)	0.152
Bladder wall interruption	11	0	42.3% (11/26)	100% (13/13)	0.61	100% (11/11)	46.4% (13/28)	0.006
Focal exophytic placental mass	10	0	38.4% (10/26)	100% (13/13)	0.58	100% (10/10)	44.8% (13/29)	0.010
Abnormal vascularization of the placental bed	17	2	65.3% (17/26)	84.6% (11/13)	0.71	89.4% (17/19)	63.6% (7/11)	0.003
≥3 MRI signs present	24	5	92.3% (24/26)	61.5% (8/13)	0.82	82.7% (24/29)	80% (8/10)	<0.001

Table legend: Se—sensibility; Sp—specificity; PPV—positive predictive value; NPV—negative predictive value.

**Table 6:** Multivariate logistic regression for the association between MRI signs and FIGO grading of PAS disorders.

MRI Signs	Grade I	*p* Value	Grade II	*p* Value	Grade IIIa	*p* Value	Grade IIIb	*p* Value
Placental bulging (OR+ CI 95%)	0.692 (0.536–0.895)	0.025	0.134 (0.15–1.221)	0.47	8.7 (1.148–65.934)	0.019	0.114 (0.045–0.287)	<0.001
Intraplacental dark T2 bands (OR+ CI 95%)	2.16 (1.2–3.89)	<0.001	0.682 (0.513–0.907)	0.010	0.794 (0.669–0.942)	0.263	0.8 (0.678–0.944)	0.323
Loss of retroplacental hypointense line on T2 images (OR+ CI 95%)	0.25 (0.003–0.247)	<0.001	9.143 (1.014–82.442)	0.025	0.735 (0.601–0.9)	0.190	0.743 (0.611–0.903)	0.248
Bladder wall interruption (OR+ CI 95%)	0.133 (0.15–1.189)	0.044	0.375 (0.82–1.714)	0.198	15.429 (1.481–160.76)	0.006	10.125 (0.922–111.247)	0.028
Focal exophytic placental mass (OR+ CI 95%)	0.615 (0.454–0.834)	0.010	0.821 (0.190–3.539)	0.791	18.667 (1.759–198.103)	0.003	3.375 (0.408–27.916)	0.239
Abnormal vascularization of the placental bed (OR+ CI 95%)	0.188 (0.041–0.851)	0.023	3.208 (0.857–12.018)	0.079	0.667 (0.099–4.508)	0.676	3.563 (0.337–37.687)	0.267

**Table 7:** Summary table presenting US and MRI signs relative incidence and true predictive value.

	Sign	Relative Incidence			True Predictive Value
		PAS Positive	PAS Negative	Total	
Ultrasonography signs	Placental lacunar spaces	92.3% (24/26)	23% (3/13)	69.2% (27/39)	88.8% (24/27)
	Loss of retroplacental clear zone	92.3% (24/26)	53.8% (7/13)	79.4% (31/39)	77.4% (24/31)
	Myometrial thinning < 1 mm	92.3% (24/26)	23% (3/13)	69.2% (27/39)	88.8% (24/27)
	Bladder wall interruption	53.8% (14/26)	0% (0/13)	35.9% (14/39)	100% (14/14)
	Bridging vessels	80.7% (21/26)	7.7% (1/13)	56.4% (22/39)	95.4% (21/22)
	Placental bulging	30.7% (8/26)	0% (0/13)	20.5% (8/39)	100% (8/8)
	Hypervascularity of the uterovesical or retroplacental space	42.3% (11/26)	7.7% (1/13)	30.7% (12/39)	91.6% (11/12)
MRI signs	Intraplacental dark T2 bands	92.3% (24/26)	61.5% (8/13)	82% (32/39)	75% (24/32)
	Placental bulging	30.7 % (8/26)	0% (0/13)	20.5% (8/39)	100% (8/8)
	Loss of retroplacental hypointense line on T2 images	96.1% (25/26)	38.5% (5/13)	77% (30/39)	83.3% (25/30)
	Myometrial thinning < 1 mm	100% (26/26)	92.3% (12/13)	97.4% (38/39)	68.4% (26/38)
	Bladder wall interruption	42.3% (11/26)	0% (0/13)	28.2% (11/39)	100% (11/11)
	Focal exophytic placental mass	38.4% (10/26)	0% (0/13)	25.6% (10/39)	100% (10/10)
	Abnormal vascularization of the placental bed	65.3% (17/26)	15.4% (2/13)	48.7% (19/39)	89.4% (17/19)

**Table 8:** Correlation between the number of US and MRI signs and surgical outcomes.

Surgical Outcomes	≥3 US Signs Present		≥3 MRI Signs Present	
	*r*	*p*	*r*	*p*
Total hysterectomy	**0.54**	**<0.001**	**0.60**	**<0.001**
Subtotal hysterectomy	0.21	0.19	−0.07	0.64
Intraoperative urinary bladder injury	0.29	0.06	**0.31**	**0.04**
Intraoperative ureteral injury	0.25	0.11	0.12	0.46
Hypogastric artery ligation	0.25	0.12	**0.34**	**0.03**
Necessity of blood transfusion	0.11	0.47	0.27	0.091
Days of hospitalization	**0.33**	**0.03**	**0.4**	**0.01**

r-correlation coefficient; bold font indicates statistical significance.

## Data Availability

The data presented in this study are available within the article.

## Supplementary Materials

The following supporting information can be downloaded at: https://www.mdpi.com/article/10.3390/diagnostics12092130/s1, Figure S1: US signs of PAS; Figure S2: MRI signs of PAS.
