# Supplementary material for: Predicting Placenta Accreta Spectrum Disorders in a Cohort of Pregnant Patients in the North-East Region of Romania—Diagnostic Accuracy of Ultrasound and Magnetic Resonance Imaging
Source: Diagnostics (Basel). 2022 Sep 1;12(9):2130. doi: 10.3390/diagnostics12092130 (PMC9497951; doi:10.3390/diagnostics12092130)
Supplement: Supplementary file 1 [file diagnostics-12-02130-s001.zip › diagnostics-1799134-supplementary.pdf]

diagnostics

MDPI

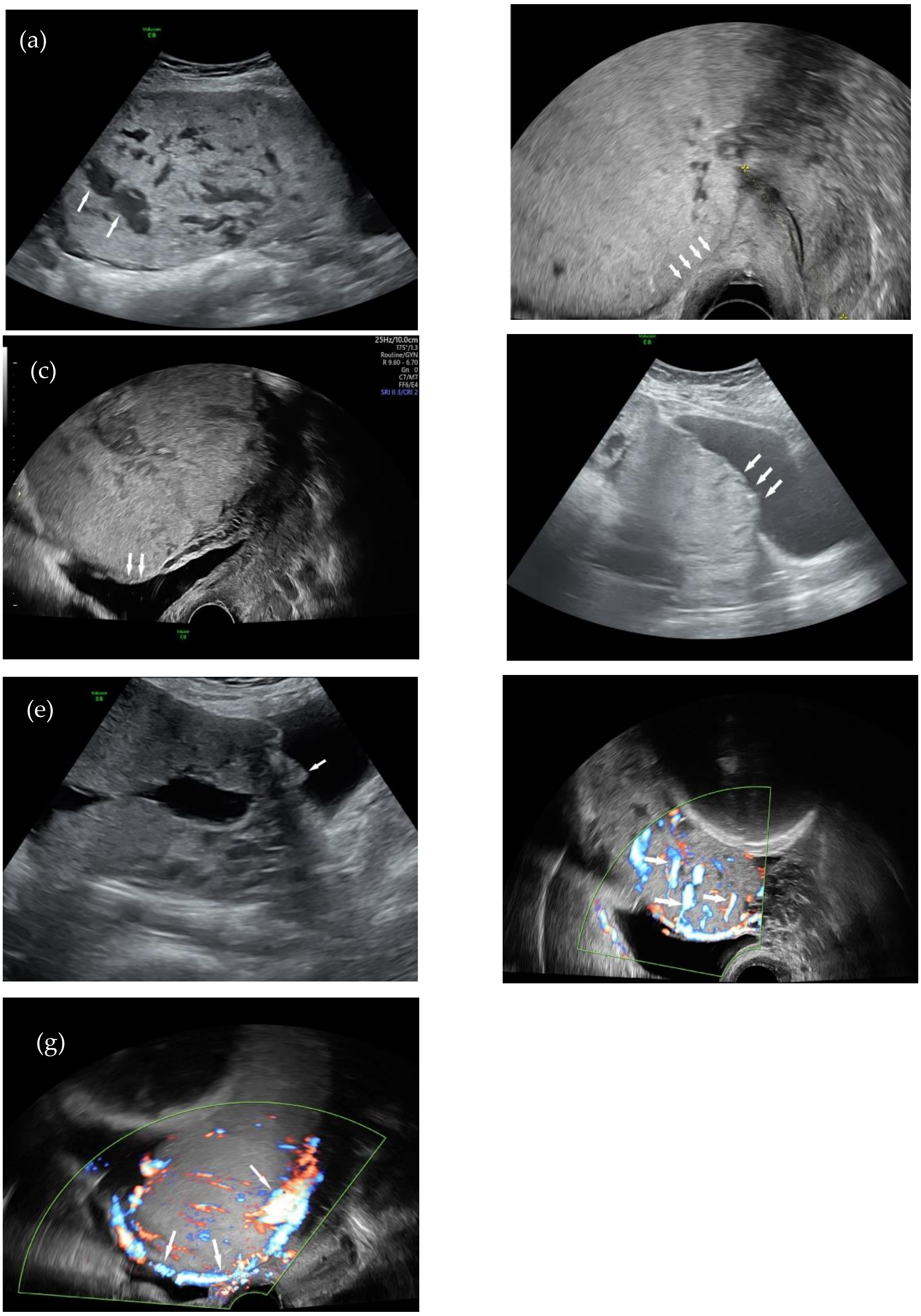

Figure S1. US signs of PAS: Grayscale (a–e) and power Doppler (f–g) images, showing the ultrasound signs used for diagnosing placenta accreta spectrum, during transabdominal or transvaginal examination. (a) Multiple irregular-shaped anechoic placental lacunae (white arrows). (b) Loss of retroplacental hypoechoic (clear) zone (white arrows). (c) Thinning of retroplacental myometrium, less than 1 mm (white arrows). (d) Bladder wall interruption (white arrow). (e) Placental bulge (white arrow). (f) Bridging vessels between placenta and bladder (white arrows). (f) Subplacental hypervascularity (white arrows).

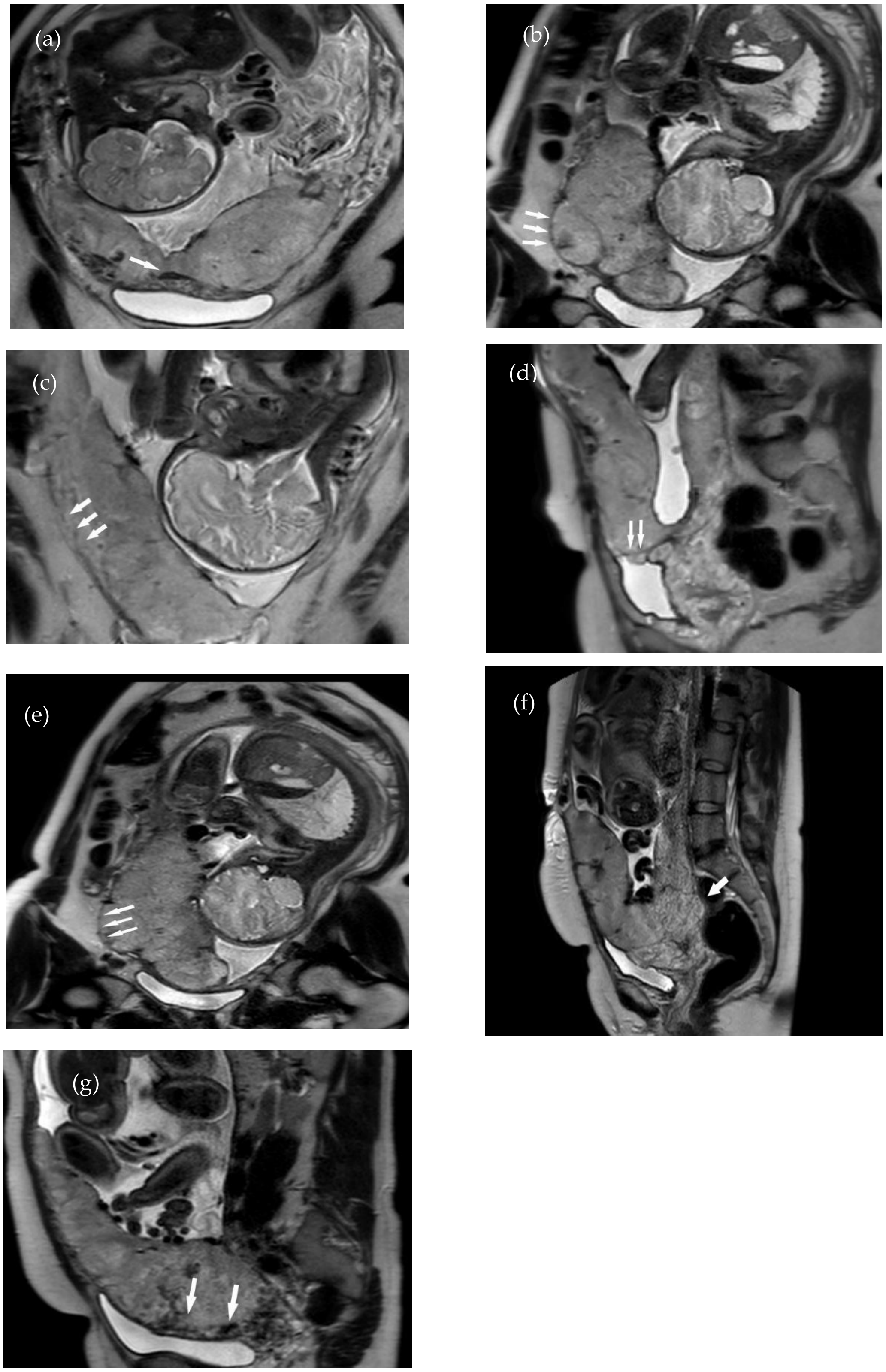

Figure S2. MRI signs of PAS: (a) Coronal T2-weighted single-shot fast spin echo (SSFSE) shows moderately heterogeneous signal intensity with abnormal intraplacental dark bands (arrows). (b) Coronal T2 SSFSE view shows a placental bulge, that appears to bulge outwards to the myometrium, causing a uterine contour deviation. (c) Coronal T2 view illustrates a loss of retroplacental dark line (arrow). (d) Bladder wall interruption in a sagital T2 weighted

view is seen as irregularity and disruption (arrow) of the normal hypointense bladder wall. (e) Myometrial thinning shown in coronal view T2 weighted sequence. (f) Sagital T2 weighted sequence showing a focal exophytic mass (arrow). (g) Retroplacental hypervascularity shown in T2 weighted sagital view (arrows).
